# Utility of the Loop-Mediated Isothermal Amplification Assay for the Diagnosis of Visceral Leishmaniasis from Blood Samples in Ethiopia

**DOI:** 10.4269/ajtmh.21-0334

**Published:** 2021-07-26

**Authors:** Dawit Gebreegzabher Hagos, Yazezew Kebede Kiros, Mahmud Abdulkader, Zekarias Gessessew Arefaine, Etsay Nigus, Henk H. D. F. Schallig, Dawit Wolday

**Affiliations:** ^1^ Mekelle University College of Health Sciences, Mekelle, Ethiopia;; ^2^ Parasitology Unit, Department of Microbiology, Amsterdam University Medical Centers, Academic Medical Center at the University of Amsterdam, Amsterdam, The Netherlands

## Abstract

Rapid and accurate diagnosis of visceral leishmaniasis (VL) is needed to initiate prompt treatment to reduce morbidity and mortality. Here, we evaluated the performance of loop-mediated isothermal amplification (LAMP) assay for the diagnosis of VL from blood in an endemic area in Ethiopia. LAMP was positive in 117/122 confirmed VL cases and negative in 149/152 controls, resulting in a sensitivity of 95.9% (95% CI: 90.69–98.66) and a specificity of 98.0% (95% CI: 94.34–99.59), respectively. The sensitivity of the LAMP assay was 95.0% (95% CI: 88.61–98.34) in HIV-negatives and 100% (95% CI: 85.18–100.0) in HIV-positives. Compared with microscopy, LAMP detected 82/87 (94.3%, 95% CI: 87.10–98.11) of the microscopy+ cases and was negative in 11/27 (40.7%, 95% CI: 22.39–61.20) of the microscopy− cases. Compared with the rK39 serology, LAMP detected 113/120 (94.2%, 95% CI: 88.35–97.62) of the rK39+ cases and was negative in 149/154 (96.8%, 95% CI: 92.59–98.94) of the rK39− cases. However, when compared with microscopy only, rK39 detected 83/87 (95.4%, 95% CI: 88.64–98.73) of the microscopy+ cases and negative in only 12/27 (44.4%, 95% CI: 25.48–64.67) of the microscopy– cases. There was an excellent agreement between rK39 and LAMP (Kappa = 0.91, 95% CI: 0.86–0.96). Furthermore, an algorithm using rK39 followed by LAMP would yield a sensitivity of 99.2% (95%CI: 95.52–99.89) and a specificity of 98.0% (95% CI: 94.34–99.59). The findings demonstrate that LAMP assay is an accurate and rapid molecular assay for VL diagnosis, including in HIV-1 coinfected patients, in an endemic setting.

## INTRODUCTION

Leishmaniasis remains one of the world’s most neglected infectious diseases (NIDs). The WHO estimates that 350 million people in 98 countries are at risk of contracting leishmaniasis and approximately 1.5 million new cases occur yearly.^1^ Around 400,000 of these are the most severe clinical manifestation, termed as visceral leishmaniasis (VL). VL, also known as Kala azar, is a devastating parasitic disease that is transmitted by the sand fly vector and kills some 95% of untreated patients. Despite improvements in treatment, diagnosis, and prevention of leishmaniasis in the last 10 years, worldwide, the disease shows a trend of geographical spread appearing in countries and areas previously free from leishmaniasis. Importantly, the emergence and spread of drug-resistant *Leishmania*, poor response to the existing drugs and relapses, and coinfection with HIV present major challenges to the control of leishmaniasis globally.

Visceral leishmaniasis is endemic in many countries in Eastern Africa, including Ethiopia, where up to 4,000 cases are reported annually, and an estimated 5 million people live at risk of being infected.^2^ Indeed, Ethiopia is one of the seven countries in which more than 90% of global VL cases occur and one of the 10 countries with the highest estimated case counts, which together account for 70–75% of global estimated VL incidence.^1^ The country has been listed by the WHO among the 14 countries in the world with the highest burden of VL.^3^ Visceral leishmaniasis, caused by *Leishmania Donovani*, is a growing public health problem in Ethiopia, with endemic areas that are continually spreading, and associated with a very high morbidity and mortality.^4^^–^^7^ HIV–*Leishmania* coinfection in Ethiopia is one of the highest (20–40%) in the world,^8^^–^^11^ though recent data suggest a trend towards reduction around 20%.^12^

Untreated VL can cause severe morbidity and ultimately leading to death in almost all cases. Accurate and prompt diagnosis of VL is thus essential to initiate treatment immediately as is monitoring of treatment efficacy to prevent morbidity and mortality. Currently, several tools for the diagnosis and monitoring of treatment efficacy for VL are under development, which is deemed key clinical research priority area in leishmaniasis according to the WHO.^13^ In several endemic areas, including Ethiopia, current gold standard diagnosis as well as monitoring of treatment efficacy for VL is based on clinical findings such as fever, organomegaly, and weight loss, confirmed by parasite detection in clinical specimens, such as bone marrow, splenic or lymph node aspirates.^14^^,^^15^ Although microscopy is widely used and has been used successfully in remote areas, its sensitivity declines with decreasing numbers of parasites, which is commonly noted in VL patients.^14^^,^^15^ In addition, aspirates for microscopy must be taken from the lymph nodes, bone marrow, or the spleen. Splenic aspirates are dangerous for the patient and the diagnostic sensitivity in other aspirates is lower than that of the spleen.^16^^–^^20^ These procedures are invasive, technically complicated and with complications and therefore difficult to perform in the most remote parts of a country where the disease is highly endemic. In addition, a range of (simple) serological diagnostic techniques have been developed that are both sensitive and cost-effective; the direct agglutination test (DAT) and detection of antibodies against the rK39 antigen are extensively used in Asia and Africa^17^^,^^18^; rK39 is also the diagnostic of choice for VL elimination from southeast Asia.^21^^–^^23^ However, antibodies detected by these tests can be found in the blood of previously infected patients for many months after the parasites have been cleared, making the detection of active infection or determining the prognosis inaccurate.^17^^,^^24^ Moreover, the sensitivity of rK39 test is significantly reduced in patients from east Africa as well as for those coinfected with HIV.^22^^,^^25^

Recently, nucleic acid amplification techniques (NAATs), such as polymerase chain reaction (PCR), have become popular choices as a tool to diagnose VL, monitor treatment response, and predict relapse because of high sensitivity and accuracy.^26^^,^^27^ The pooled sensitivity of PCR in whole blood was 93.1% (95% CI, 90.0–95.2), and the specificity was 95.6% (95% CI, 87.0–98.6). PCR for patients with HIV-VL coinfection showed high diagnostic accuracy in buffy coat and bone marrow, ranging from 93.1% to 96.9%. Likewise, techniques such as real-time–PCR (RT-PCR) have become available for parasite quantification.^28^^,^^29^ Although these techniques have sufficient sensitivity and ability to detect active infections, they also require expert technical skill, rather sophisticated equipment, continuous electricity supply, and are considerably more expensive than the serological tests. Therefore, many of these tests are not suitable for routine use in endemic regions, except in well-equipped reference laboratories. Isothermal amplification techniques such as nucleic acid sequence-based amplification (NASBA) for leishmaniasis have been developed as alternative NAAT,^29^^,^^30^ but this technology targets RNA requiring rather specific nucleic acid extraction.

Despite progress in the development of diagnostics and treatment monitoring during the last two decades, to date, there is no reliable rapid molecular test that is highly sensitive and specific for the diagnosis of VL. Some of the few recent advantages in diagnostic test development may open ways to introduce NAATs in VL case management near patient. The first technology is loop-mediated isothermal reaction (LAMP),^31^ which uses only one enzyme (*Bst* DNA polymerase) and is able to amplify large amounts of DNA within 30–60 minutes by the intricate design of primers and auto-strand displacement DNA synthesis. The reaction has several advantages: firstly, the reaction takes place between 60°C and 65°C, and can therefore be completed without the use of a thermocycler. Secondly, the specificity of the reaction is high because of the design of six primers. Thirdly, the product can be visualized directly using simple detection methods. Also, the stability of LAMP reagents was studied and showed to be unaffected by ambient temperatures up to 37°C, potentially negating a cold-chain process.^32^ Therefore, LAMP has emerged as a powerful diagnostic tool for several NIDs and has been implemented for important protozoan parasitic diseases such as malaria,^33^^,^^34^ and trypanosomiasis.^32^^,^^35^^,^^36^ Importantly, LAMP has also been developed for the diagnosis of VL in Asia.^37^^–^^41^ There is limited data, however, on the utility of LAMP assay for VL diagnosis in the African setting, including HIV-1 coinfected patients.^42^^–^^44^

Overall, the development of rapid molecular tests for the diagnosis of VL is showing significant progress, the need for the evaluation of novel diagnostic tools in endemic areas remains top priority, including in HIV coinfected patients. Here, we aimed to evaluate the utility of the LAMP assay as a diagnostic tool for VL in an endemic setting in northern Ethiopia.

## METHODS

### Study site and participants.

This study was undertaken between June 2019 and December 2020 at Ayder Generalized Referral Hospital, affiliated to Mekelle University College of Health Sciences, Mekelle City, in Tigrai Regional State, northern Ethiopia. The study is part of the European and Developing Countries Clinical Trial Partnership (EDCTP)-funded EvaLAMP project, a prospective cohort study evaluating the utility of loop-mediated isothermal amplification assay (LAMP) for VL diagnosis (Clinicaltrials.gov: NCT04003532).

Patients with clinical signs and symptoms suggestive of VL (including fever > 2 weeks, weight loss, splenomegaly, hepatomegaly, or lymphadenopathy^45^) were enrolled prospectively in the study. Patients were excluded if they provide a treatment history with any antileishmanial drugs within the previous 3 months, not capable of understanding or complying with the study protocol, or refusal to consent and participate in to the study. Suspected VL cases were tested using the rK39 rapid diagnostic test (RDT) and microscopic examination of Giemsa-stained splenic or bone marrow aspirates for the presence of *L. donovani* amastigotes.

In addition, we enrolled endemic healthy controls (EHCs) as well as nonendemic healthy controls (NEHCs). EHCs were recruited from the same hospital from individuals who were rK39 negative and microscopy negative cases. NEHCs were specimens from subjects with no travel history in to a VL endemic area.

### Sample size.

The sample size required for the evaluation of the LAMP assay, based on an expected sensitivity of 90% and specificity of 90%, a desired binomial error margin of ± 5% and an α level of 0.05 was estimated to be 62 VL cases and 62 controls. With an expected 50% prevalence of VL among suspects, a minimum of 124 VL suspects should be screened to identify 62 confirmed VL cases and 62 controls. Taking into account an anticipated response rate of 80%, we aimed to recruit a minimum total sample size of 156 VL suspects.

### Reference standard VL diagnosis.

The reference standard for the diagnosis of VL included parasite identification from tissue aspirates or biopsy on Giemsa-stained specimens and/or rK39 positive RDT results from subjects suspected of VL.^41^

### LAMP assay.

Whole blood samples were processed by boil and spin method.^44^ In brief, 95 µL whole blood were mixed with 5 µL 10% sodium-dodecyl sulfate by inversion 10 times in a 1.5-mL Eppendorf tube, then allowed to stand for 10 minutes at room temperature and mixed again. After adding 400 µL of PCR grade H_2_O, the mixture was incubated in a heating block at 90°C for 10 minutes, and then centrifuged at 13,000 rpm for 3 minutes. The supernatant was then aspirated and analyzed immediately.

**Figure 1.**
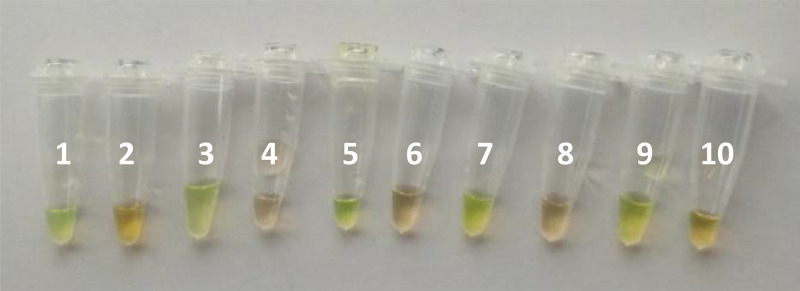
Loop-mediated isothermal amplification (LAMP) assay results. Tube 1: positive control; tube 2: negative control; tubes 3, 5, 7, and 9 are positive visceral leishmaniasis (VL) samples; tubes 4, 6, 8, and 10 are non-VL samples. This figure appears in color at www.ajtmh.org

The LAMP assay was done using the Loopamp *Leishmania* Detection Kit (Eiken Chemical Co., Tokyo, Japan). The Loopamp *Leishmania* Detection Kit uses primers that target two different regions of the *Leishmania* genome: the 18S rRNA gene and the kDNA minicircles, which are specific to the *Leishmania* genus. For amplification, Bst DNA polymerase is used, and the dried reagents include calcein to allow for the visual judgment of the amplified products without opening the reaction tubes. The kit includes negative and positive controls; the positive control is an artificial construct based on DNA sequences from *L. donovani* isolates (GenBank Accession Numbers Y11401 and X07773). Three µL of the DNA obtained by the DNA extraction method described earlier was used for the LAMP reaction. This was run for 40 minutes at 65°C in the LF-160 incubator (HumaLoop M incubator, HUMAN, Wiesbaden, Germany). Positive samples emit a green fluorescent light and negative samples do not emit any light (Figure 1). LAMP assay was done by investigators who were blinded from the results of all other tests as well as clinical data of VL suspects.

### rK39 serology.

For all the subjects, rK39 RDT was performed from plasma samples according to manufacturer’s instruction (IT LEISH, Bio-Rad Laboratories, The Netherlands).

### HIV serology.

Screening for HIV-1 was done using RDT based on National Guidelines.^46^ Screening for HIV-1 was done using STAT-PACK (Chembio Diagnostic Systems Inc., New York, NY); those positive were then confirmed by a second test ABON (Abon Biopharma, Hangzhou, China), and in the event of discrepant results, a tie-breaker with SD Bioline (SD Standard Diagnostics Inc., Seoul, South Korea) was done.

### Statistical analysis.

Baseline characteristics for continuous variables were expressed as the median with interquartile range (IQR), and for categorical variables as proportions. The χ^2^ test was used to compare categorical variables and Wilcoxon rank-sum test for not normally distributed variables.

The performance of the LAMP assay was compared with the reference standard diagnosis for VL based on parasite identification and/or rK39 serology, as described previously.^41^ Agreement between different tests was calculated using Cohen’s kappa coefficient.^47^ Moreover, we also determined the performance of the LAMP assay against microscopy alone or rK39 RDT assay, as these tests are being used routinely for the diagnosis of VL in resource-constrained settings. We also assessed the performance of a diagnostic algorithm where rK39 and LAMP would be used sequentially to simplify and improve the diagnosis of VL in the context of resource-poor settings. A test was considered significant if *P* value was < 0.05. Reporting of the diagnostic performance of the tests in this study was according to the Standards for Reporting of Diagnostic Accuracy Studies (STARD) guidelines.^48^ Statistical analysis was carried out using STATA statistical software version 14.0 (StataCorp, College Station, TX).

### Ethical considerations.

The study protocol was reviewed and approved by the Health Research Ethics Review Committee (ERC#: 1102/2017), Mekelle University College of Health Sciences and the National Research Ethics Review Committee, Ministry of Science and Higher Education. Informed consent was obtained from all study participants or guardians.

## RESULTS

### Clinical samples.

Two hundred seventy-four individuals with clinical signs and symptoms compatible with VL were enrolled, of whom 122 were confirmed VL cases (83 were microscopy+ and rK39+; 4 microscopy+ and rK39−, and 35 were microscopy− and rK39+). Sociodemographic and clinical characteristics of the VL cases are shown in Table 1. Of all the VL cases, 23 (18.9%) were also coinfected with HIV. Baseline sociodemographic data is summarized in Table 1. Of the 122 confirmed VL cases, the majority were male (95.1%). The median age of the VL patients was 23 years (IQR: 18–32). All fulfilled the clinical case definition for VL and we did not find any significant differences in either clinical or laboratory parameters between those who were microscopy+ and rK39+ cases. In addition, a total of 152 controls were enrolled, of whom 50 comprised HECs and 102 were NEHCs.

**Table 1:** Sociodemographic characteristics of study participants (VL cases)

Characteristic	Overall	Microscopy+	rK39+
	(*N* = 122)	(*N* = 87)	(*N* = 118)
Age (median years [IQR])	23 (18–32)	24 (19–31)	24 (19–32)
Sex			
Male	116 (95.1)	84 (96.6)	112 (94.9)
Female	6 (4.9)	3 (3.4)	6 (5.1)
VL symptoms			
Fever (> 2 weeks)	119 (97.5)	86 (98.9)	115 (97.5)
Weight loss	118 (96.7)	85 (97.7)	114 (96.6)
Fatigue	119 (97.4)	84 (96.6)	115 (97.5)
Abdominal swelling	109 (89.3)	80 (92.0)	105 (89.0)
Clinical signs			
Temp. (°C, median IQR)	38.1 (37.2–38.7)	38.1 (37.5–38.9)	38.1 (37.2–38.7)
Splenomegaly	113 (92.6)	81 (93.1)	109 (92.4)
Hepatomegaly	50 (41.0)	30 (34.5)	48 (40.7)
Laboratory markers			
WBC (×10^9^/L)	1.7 (1.2–2.1)	1.6 (1.1–2.0)	1.7 (1.2–2.1)
Hemoglobin (mg/dL)	7.3 (6.2–8.5)	7.3 (6.3–8.5)	7.3 (6.2–8.5)
Platelet count (×10^9^/L)	117 (71–240)	98 (56–180)	117 (72–250)
ALT (U/L)	65 (45–142)	63 (51–125)	67 (44–146)
ALT (U/L)	39 (25–58)	38 (26–50)	39 (25–73)
Alkaline phosphate (U/L)	163 (88–308)	154 (114–178)	166 (112–308)
Bilirubin-direct (mg/dL)	0.44 (0.31–1.31)	0.50 (0.34–1.31)	0.44 (0.31–1.31)
BUN (mg/dL)	23 (15–28)	20 (15–27)	22 (15–28)
Creatinine (mg/dL)	0.62 (0.45–0.80)	0.62 (0.44–0.73)	0.60 (0.45–0.79)

AST = aspartate aminotransferase; ALT = alanine aminotransferase; BUN = blood urea nitrogen; IQR = interquartile range; WBC = white blood cell. Data are either proportions (%) or median (25–75% interquartile).

### Performance characteristics of the LAMP assay.

The performance characteristics of the LAMP assay is summarized in Table 2. Among the 122 confirmed VL cases, 117 were positive with LAMP and of the 152 controls, 149 were negative, resulting in a sensitivity of 95.9% (95% CI: 90.69–98.66) and a specificity of 98.0% (95% CI: 94.34–99.59). All 23 VL cases coinfected with HIV-1 were positive with LAMP, yielding a sensitivity of 100% (95% CI: 85.18–100.0). The sensitivity of the LAMP assay in HIV-1 negative VL patients was 95.0% (95% CI: 88.61–98.34).

**Table 2:** Performance of the LAMP diagnostic assay for the diagnosis of visceral leishmaniasis in clinical samples, northern Ethiopia

Sample group	Cases tested	Cases positive for LAMP	Sensitivity/specificity (95% CI)
Visceral leishmaniasis			
All cases	122	117	95.9 (90.69–98.66)
HIV uninfected	99	94	95.0 (88.61–98.34)
HIV coinfected	23	23	100.0 (85.18–100.0)
Controls			
All controls	152	3	98.0 (94.34–99.59)
Endemic-healthy controls	50	1	98.0 (89.35–99.95)
Nonendemic healthy controls	102	2	98.0 (93.10–99.76)

LAMP = loop-mediated isothermal amplification.

### Comparison of different assays.

When compared with the microscopic exam alone, LAMP assay detected 82/87 (94.3%, 95% CI: 87.10–98.11) of the microscopy+ VL cases and was negative in 11/27 (40.7%, 95% CI: 22.39–61.20) of the microscopy− cases. When compared with the rK39 RDT alone, LAMP assay detected 113/120 (94.2%, 95% CI: 88.35–97.62) of the rK39+ cases and was negative in 149/154 (96.8%, 95% CI: 92.59–98.94) of the rK39− cases. However, when compared with the microscopic exam alone, rK39 detected 83/87 (95.4%, 95% CI: 88.64–98.73) of the microscopy+ VL cases and was negative in only 12/27 (44.4%, 95% CI: 25.48–64.67) of the microscopy− VL suspects. Although we noted an excellent agreement between rK39 and LAMP (Kappa = 0.91, 95% CI: 0.86–0.96), the agreement between LAMP and microscopy (Kappa = 0.41, 95% CI: 0.20–0.61), and between rK39 and microscopy (Kappa = 0.46, 95% CI: 0.26–0.66) were moderate.

Finally, we assessed the performance of a diagnostic algorithm where rK39 and LAMP would be used sequentially.^44^ As noted in Table 3, of the total 122 confirmed VL cases, the algorithm would yield a sensitivity of 99.2% (95%CI: 95.52–99.89). Furthermore, of the total 152 controls, the algorithm would yield a specificity of % 98.0 (95% CI: 94.34–99.59).

**Table 3:** Performance of the rK39-LAMP algorithm for the diagnosis of visceral leishmaniasis in clinical samples, northern Ethiopia

Sample group	Cases tested	Cases positive for LAMP	Sensitivity/specificity (95% CI)
Visceral leishmaniasis	122	121	99.2 (95.52–99.89)
Controls	152	3	98.0 (94.34–99.59)

LAMP = loop-mediated isothermal amplification.

## DISCUSSION

Very little is known with respect to the clinical utility of the LAMP assay for the diagnosis of VL in the African setting, in particular among HIV-1 coinfected patients. Previous study on LAMP for *L. donovani* diagnosis in Africa used was based on *Leishmanial* 18sDNA and demonstrated a sensitivity of 83% and specificity of 98% when compared with microscopy of bone marrow and lymph node aspirate samples.^42^ The performance of the assay was, however, improved to a sensitivity of 92.3% and specificity of 100% when Qiagen extraction method was used.^43^ Furthermore, a recent study conducted in the Sudan demonstrated a better sensitivity (100%) and specificity (99%) when using whole blood specimens and Qiagen extraction method and optimizing primer design.^44^ All the studies conducted in Africa so far are limited because of small sample size. In addition, differences in methods used, including DNA extraction and nature of the specimen,^43^^,^^44^^,^^49^ as well as parasite heterogeneity^50^ might all have contributed to the differences in the performance of the LAMP assay. Differences in performances of the LAMP assay in different studies might be also attributed to the differences in the target genes used for the amplification.^49^ The manufacturer of the Loopamp^™^ Leishmania Detection kit reported a sensitivity of 85% and a specificity of 97%. In the current study, we observed a sensitivity of 95.9% and a specificity of 98.0%. The high sensitivity and specificity shown in our study concurs with recent findings reported from Ethiopia and the Sudan, notably related to the use of both 18S rRNA and the kDNA target genes.^43^^,^^44^ An additional reason for differences may ensue also due to the use of different reference standards. Whereas in most of the studies undertaken that evaluated the performance of the LAMP assay, microscopy was the reference test used,^37^^,^^38^^,^^42^^–^^44^ only few used additional PCR as reference standard.^39^^–^^41^ In this study, the composite reference standard we used was a combination of microscopy and rK39 RDT, that was similar to the report by Dixit et al.^41^

The only study that evaluated LAMP among HIV-VL coinfected cases is the one reported by Adams et al.^43^ Albeit having small number of coinfected patients (*N* = 7), they noted a sensitivity of 100%. The findings concur with ours where we demonstrated a sensitivity of 100% among 23 VL patients coinfected with HIV suggesting that LAMP assay is also optimum for the diagnosis of VL in coinfected patients.

Mukhtar et al.^44^ suggested an algorithm by adding the LAMP assay for improving the diagnosis of VL. However, the sensitivity and specificity of such an algorithm for improving the diagnosis of VL has not been done previously. Thus, we also assessed the performance of a diagnostic algorithm where rK39 and LAMP would be used sequentially to simplify and improve the diagnosis of VL in the context of resource-poor settings. In the current study, the algorithm yielded better performance with a sensitivity of 99.2% and a specificity of 98.0% when compared with rK39 RDT serology alone.

The strength of the current study is that it is a well-designed prospective nature of the study and including well-characterized controls (EHCs and NEHCs). Another strength of the current study is the use of a reference standard criteria for active VL, that is, positive by microscopy and/or rK39 RDT serology, as noted in previous study.^41^ The reason why we added rK39 in addition to parasitology is the fact that parasitology, although highly specific, is compounded by low levels of sensitivity have been reported from endemic areas that depended also on the nature of the specimen.^14^^–^^20^ Indeed, the sensitivity of microscopy is around 54–65%, 60–85%, and 90%, for lymph node, bone marrow, and splenic aspirate specimens, respectively.^18^^–^^20^ However, limitation of the study is lack of comparable results with DAT.

## CONCLUSION

Overall, the Loopamp Leishmania Detection Kit has shown a very good diagnostic performance in this study, and the possibility of using a simple sample preparation method (Boil and Spin) and a portable and robust real-time fluorimeter opens an avenue for the diagnosis of VL at the PoC, enabling treatment when confirmatory diagnosis is required. This test can support the diagnosis of VL in situations in which serological diagnosis is useless such as in VL relapses and test-of-cure, VL/HIV coinfection, and the diagnosis of leishmaniasis cases that require confirmatory diagnosis to initiate systemic treatment, which often presents high toxicity. The current cost of the Loopamp Leishmania Detection kit is around 8 US$ per test. This cost is expensive compared with currently available tests in our setting (for microscopy, or rK39 RDT it is less than 1 US$). Provided that there will be an increase in the demand for its future use in endemic regions, it is presumed that the cost of the Loopamp Leishmania Detection kit is expected to decrease substantially through various access modalities.
